# Epithelial Cyst in the Posterior Triangle of the Neck: Atypical Branchial Cyst or Cystic Lymph Node Metastasis?

**DOI:** 10.1155/2014/912347

**Published:** 2014-01-12

**Authors:** Domenic Vital, Gerhard F. Huber, Thomas F. Pézier, Matthias Rössle, Rudolf Probst, Gian-Marco Widmer

**Affiliations:** ^1^Department of Otorhinolaryngology, Head and Neck Surgery, University Hospital of Zurich, Frauenklinikstrasse 24, 8091 Zurich, Switzerland; ^2^Institute of Surgical Pathology, University Hospital of Zurich, Schmelzbergstrasse 12, 8091 Zurich, Switzerland

## Abstract

We report the case of a 66-year-old man with a cervical neck mass located behind the left sternocleidomastoid muscle. To exclude malignancy, a full workup, including clinical, radiological, and cytological examination, was performed but failed to provide a definitive diagnosis. Histological analysis following excisional biopsy revealed a benign epithelial cyst, consistent with an atypically located branchial cyst. We describe an approach to the management of these neck masses and discuss several theories of the etiology of branchial cysts and how they may come to be abnormally located.

## 1. Case Presentation

**Figure 1:**
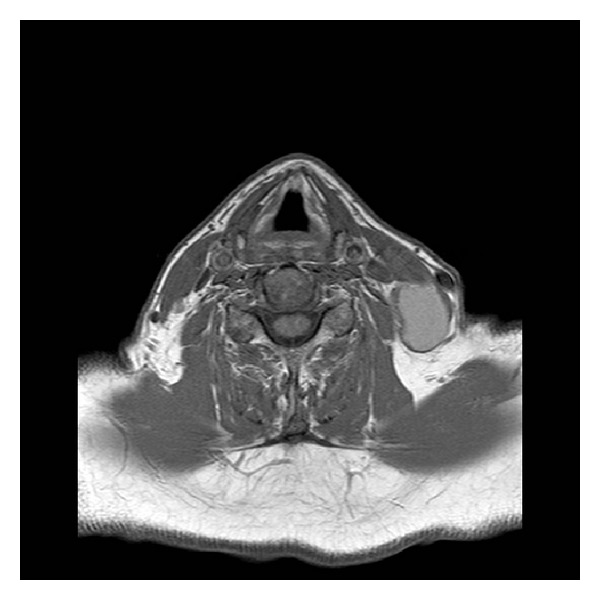
Axial slides of T1-weighted MRI showing a hyperintense cystic lesion behind the left sternocleidomastoid muscle in the neck level five.

**Figure 2:**
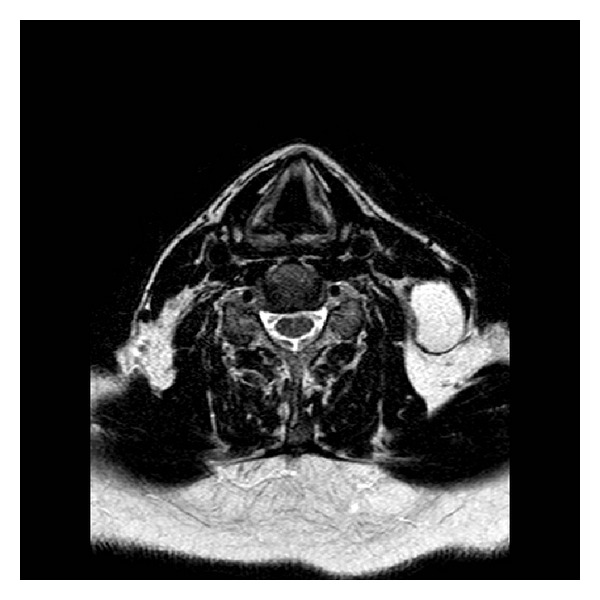
Axial slides of T2-weighted MRI showing a hyperintense cystic lesion behind the left sternocleidomastoid muscle in the neck level five.

A 66-year-old man was referred to our department with a 2-day history of a painless left cervical neck mass. He denied any other symptoms and reported that he only occasionally drank alcohol and had stopped smoking some 30 years ago with a total of 10 pack years. Clinical examination revealed a well-defined, painless neck lump, posterior to the sternocleidomastoid muscle close to the mastoid. The lump was some 5 centimeters in size, roughly oval, and not fixed to adjacent structures. There were no surrounding skin changes or other associated findings. Intraoral examination and transnasal fiber-endoscopy were normal. Cervical ultrasound showed an irregularly walled mass, 5 cm in diameter, with hyperechogenic reflections in an echo-poor center. The other neck structures were sonographically normal. Magnetic resonance imaging (MRI) showed a cystic lesion with an irregular wall lateral and posterior to the sternocleidomastoid muscle (neck level five; see Figures 1 and 2). Repeated fine-needle aspiration cytology (FNAC) revealed squamous epithelial cells without signs of malignancy, consistent with a branchial cleft cyst. However, given the patient's age and the atypical localization, the differential diagnosis included a metastasis of a well-differentiated squamous cell carcinoma (SCC). We therefore recommended the patient to undergo panendoscopy and extirpation of the mass with intraoperative frozen section analysis of the specimen. The patient agreed with neck dissection in case of malignancy. Panendoscopy revealed no extra findings, and both intraoperative frozen section and definitive histology of the excisional biopsy confirmed a branchial cleft cyst. The patient recovered well after surgery and was discharged from follow up 12 months later.

## 2. Discussion

In patients older than 40 years, especially with risk factors for malignant disease, it is prudent to consider all cystic lesions of the neck as malignant until proven otherwise. In patients younger than 40 years, clinicians should be aware of a metastasis of a papillary thyroid carcinoma [1]. After a careful history and thorough clinical examination further investigations should include ultrasonography of the neck including FNAC and 3-dimensional imaging either with computed tomography (CT) or MRI. Ultrasound-guided FNAC is often diagnostic but its sensitivity drops from >95% in solid tumors [2–4] to 50%–73% in cystic lesions [5–7]. If FNAC fails to provide a definitive diagnosis, we recommend further diagnostic steps including panendoscopy of the upper aerodigestive tract and excision of the cyst with intraoperative frozen section.

In terms of malignant lesions, squamous cell carcinoma (SCC) of Waldeyer's ring (e.g., palatine and lingual tonsils) and papillary thyroid cancers have a predilection for cystic lymph node metastases [2, 8, 9]. However, the exact mechanism for the development of cystic lymph node metastases is unclear. Tumor necrosis forming a pseudocyst has been found as well as true cystic cavities lined by neoplastic epithelium. Indeed, the expression of cytokeratin 7 has led some to hypothesize that a subset of SCC of Waldeyer's ring might originate from excretory ducts of minor salivary glands and therefore show the tendency to form cystic lesions [10].

The aetiology of benign cervical cysts is also unclear. The most popular, but still controversial, theory is the branchial apparatus theory first described by Von Ascherson in 1832. Unfortunately, atypical locations of branchial cysts are poorly explained by this theory, and alternatives have been proposed such as the cervical sinus theory, the thymopharyngeal theory, and the inclusion theory [11–13]. The cervical sinus theory is an extension of the branchial apparatus theory focusing on lateral cervical cysts. It considers that branchial fistulae were related to the cervical sinus rather than the pharyngeal clefts and pouches. Other authors hypothesized that the development of lateral cervical cysts is linked to the embryology of the thymus, which originates from the third pharyngeal pouch via the thymopharyngeal duct.

Consequently, the thymopharyngeal theory indicates that lateral cervical cysts are a result of an incomplete obliteration of the thymopharyngeal duct [14].

Reporting a similar case to our own, Grignon et al. [15] proposed that the location of the branchial cyst was the result of the organogenesis of the sternocleidomastoid muscle, which is independent of the development of the branchial apparatus and proceeds in a craniocaudal and dorsoventral fashion. It therefore would predict that cranial branchial cysts would be located anteriorly to the muscle, and a caudal branchial cyst posteriorly [15]. Bhaskar and Bernier [13] examined 468 specimens of branchial cysts and proposed that most of the branchial cysts represent cysts in lymph nodes, which originated from inclusion of epithelium during embryogenesis (so-called “inclusion theory”). Their conclusions helped to explain three findings which seem inconsistent with Grignon's theory: (1) the presence of surrounding lymphoid tissue such as sinusoids in the cysts, (2) size fluctuations during infections of the upper aerodigestive tract, and (3) the fact that the cysts are hardly ever seen at birth. Consequently, it was proposed to use the term *“benign lymphoepithelial cyst”* instead of *“branchial cyst”* [13]. Furthermore, this theory also explains the rare cyst location posterior to the sternocleidomastoid muscle. Indeed, in their series, Bhaskar and Bernier found 5 of the 468 cysts located in the posterior part of the neck triangle (neck level five) [13].

## 3. Conclusion

The diagnosis of a branchial cyst should—especially in patients older than 40 years—only be considered once malignancy has been excluded and should not be discounted because of an atypical location. Whilst in older patients metastatic SCC is more likely, in younger patients metastatic papillary thyroid cancer should be considered. Several theories exist as to the aetiology of branchial cyst formation, and some, such as the inclusion theory, are better able to explain abnormal locations.
